# Effects of Gellan Gum and Homogenization Pressure on the Stability of Milk-Based Cocoa Beverages

**DOI:** 10.3390/foods15213832

**Published:** 2026-10-26

**Authors:** Saniye Akyıl Öztürk, Deniz Damla Altan Kamer, Necattin Cihat Icyer, Atefeh Karimidastjerd, Ibrahim Palabiyik, Senay Simsek, Omer Said Toker

**Affiliations:** 1 Food Engineering Department, Chemical and Metallurgical Engineering Faculty, Yildiz Technical University, Istanbul 34220, Türkiye; 2 Azelis Application and Training Center, Istanbul 34771, Türkiye; 3 Department of Food Engineering, Faculty of Agriculture, Tekirdag Namik Kemal University, Tekirdag 59030, Türkiye; 4 Department of Food Engineering, Faculty of Engineering and Architecture, Muş Alparslan University, Muş 49250, Türkiye; 5 Whistler Center for Carbohydrate Research, Department of Food Science, Purdue University, West Lafayette, IN 47907, USA

**Keywords:** gellan gum, milk-based cocoa beverages, molecular docking, sedimentation prevention, beverage stability

## Abstract

Milk-based cocoa beverages are susceptible to physical instability, particularly sedimentation and creaming, because of the poor dispersibility and structural characteristics of cocoa particles. This study investigated the synergistic effects of gellan gum concentration and homogenization pressure on beverage stability using a Central Composite Rotatable Design. We evaluated water-holding capacity, sedimentation, creaming, pH, viscosity, and torque, and compared the optimized formulation with a commercial cocoa milk product. Particle size distribution, zeta potential, Fourier-transform infrared spectroscopy, optical microscopy, scanning electron microscopy–energy-dispersive spectroscopy, molecular docking, and sensory analysis were used to characterize the physicochemical, structural, molecular, and sensory properties of the beverages. The optimum conditions were identified as 0.04% gellan gum and a homogenization pressure of 214.82 bar. Under these conditions, the beverage exhibited a high water-holding capacity (93%), no detectable sedimentation, minimal creaming (0.012%), an apparent viscosity of 59.44 mPa·s, a reduced mean particle size of 309.9 nm, and a zeta potential of −38.73 mV. Molecular docking further supported the stabilizing role of gellan gum through hydrogen bonding, hydrophobic interactions, and electrostatic interactions with major milk proteins. The optimized beverage also, showed acceptable sensory properties and also, better physicochemical stability compared with the commercial product during nine months of refrigerated storage. Overall, the combined use of gellan gum and optimized homogenization pressure provides an effective strategy for producing stable, homogeneous, and high-quality milk-based cocoa beverages for industrial application.

## 1. Introduction

Milk-based cocoa beverages are widely consumed colloidal systems containing fat droplets, proteins, carbohydrates, and dispersed insoluble particles, including cocoa solids and dietary fiber. The physicochemical characteristics of cocoa particles can create major stability challenges in dairy matrices, including sedimentation, agglomeration, and the formation of visibly distinct light and dark layers during storage [1,2,3]. These defects reduce product uniformity and may negatively affect consumer acceptance. Hydrocolloids are therefore commonly incorporated into cocoa-based beverages to improve physical stability by limiting flocculation, coalescence, creaming, and phase separation [4]. Their stabilizing effect is consistent with Stokes’ law, which states that increasing continuous-phase viscosity and reducing dispersed-particle size can decrease gravitational separation in colloidal systems [2,5]. Hydrocolloids also interact with water and form structured networks within the liquid phase, thereby enhancing suspension stability. Gellan gum, pectin, and carrageenan are among the most frequently used stabilizers in cocoa-based beverages, although their performance depends on their molecular characteristics and interactions with the food matrix [3,6].

Several interrelated factors influence the stability of cocoa and other flavored milk beverages. One major factor is variation in milk protein content, which can result from seasonal changes and animal-feeding practices [7]. This variability challenges manufacturers seeking consistent product quality and may require adjusting stabilizer type and concentration based on the protein composition of the milk base [8]. Cocoa properties are also important. Natural cocoa powder is hydrophobic and moderately acidic, with a typical pH of 5.2–5.6, whereas alkalized or Dutched cocoa is commonly used in beverage formulations because alkaline treatment increases its pH toward neutrality, generally to approximately 7.0–8.0 [9]. In beverages containing less than 3% protein, carrageenan may not form sufficiently strong associations with casein to prevent sedimentation [10,11]. In contrast, gellan gum has been reported to provide stronger stabilization than κ-carrageenan in low-protein beverages and to reduce sedimentation more effectively [10,12,13]. Gellan gum can also form a stabilizing network that is less dependent on casein concentration, making it comparatively robust to variations in milk protein content [14,15].

A second important factor is the type and concentration of hydrocolloid used, because these variables influence not only physical stability but also viscosity and mouthfeel [11]. Carrageenan stabilizes dairy systems through interactions with protein domains; however, its performance can be limited in low-protein beverages [10]. Although carrageenan is widely used as a food additive, concerns have been raised regarding its potential association with intestinal inflammation in some animal models [16]. Nevertheless, the Joint FAO/WHO Expert Committee on Food Additives has considered its use at levels up to 1000 mg/L in infant formula to be safe [17,18]. A third factor is the physical nature of the added flavoring material, including cocoa, chocolate, strawberry, or banana powders, because particle size, density, surface properties, and dispersibility directly influence sedimentation and phase separation.

Gellan gum is an anionic microbial polysaccharide produced by *Sphingomonas elodea*. Its backbone consists of a linear tetrasaccharide repeating unit composed of 1,3-β-D-glucose, 1,4-β-D-glucuronic acid, 1,4-β-D-glucose, and 1,4-α-L-rhamnose [19]. Based on acyl content, gellan gum is generally classified as high-acyl or low-acyl. In the high-acyl, or native, form, acetate and glycerate substituents are present on the same glucose residue, with approximately one glycerate group per repeating unit and one acetate group per two repeating units. Low-acyl gellan gum is produced by alkaline treatment at elevated temperatures, which removes the acyl substituents [20]. These structural differences strongly influence gel behavior. High-acyl gellan forms soft, elastic, and non-brittle gels, whereas low-acyl gellan forms firmer, less elastic, and more brittle gels [6,21].

The textural and rheological properties of gellan gum systems can be tailored by adjusting polymer concentration and formulation composition. Depending on processing conditions and interactions with other ingredients, gellan gum can form structures ranging from weak fluid gels to firm, brittle networks. This versatility has made it a valuable hydrocolloid for a wide range of food applications [12,14,15]. In milk-based cocoa beverages, gellan gum is particularly useful because it can suspend insoluble cocoa particles and emulsion droplets even at relatively low apparent viscosity [13]. Its safety profile also supports its broader use in foods. Toxicological studies have shown low acute toxicity, no evidence of genotoxicity or carcinogenicity, and no adverse effects in subchronic studies at the highest tested doses. Human consumption at doses up to 200 mg/kg body weight per day for three weeks was also reported without adverse effects [22]. Accordingly, no numerical acceptable daily intake has been considered necessary for gellan gum (E 418), and no safety concern has been identified for its authorized use as a food additive [22].

In milk systems, high-acyl gellan gum can form self-supporting, non-syneresing gels at concentrations above approximately 0.2%, whereas much lower concentrations, typically 0.02–0.05%, produce weak, liquid-like fluid gels with measurable yield stress. These weak networks are particularly useful for suspending particles while maintaining a drinkable texture. High-acyl gellan can stabilize a broad range of protein-containing beverages, including dairy-, vegetable-, cereal-, and nut-based systems, without substantially compromising mouthfeel. It also hydrates under conventional HTST and UHT processing conditions and rapidly develops structure during cooling, providing good processing tolerance and compatibility with low-protein, reconstituted, and whey-substituted dairy matrices. Because its gelation is relatively independent of milk proteins, it can also be blended with other hydrocolloids or emulsifiers to tailor texture and sensory properties. By contrast, low-acyl gellan forms thermoreversible milk gels primarily through ionic interactions, and controlled calcium addition can improve its thermal stability. However, calcium addition must be carefully managed, generally at approximately 55–65 °C, to minimize the risk of protein precipitation. In reconstituted milk systems, milk powders can also act as natural ion-sequestering agents, reducing the need for additional chelators except under conditions involving low milk-solids content or very hard water [23]. The stabilization of cocoa milk can be considered a multifactorial process involving homogenization, hydrocolloid-mediated structuring, milk protein–polysaccharide interactions, and the electrochemical environment of the dispersed system. Homogenization can reduce the size of dispersed fat droplets and particle aggregates and promote the formation of protein-covered interfaces, thereby reducing the tendency of the dispersed phase to separate. In parallel, hydrated gellan gum can increase the structural organization of the continuous phase and restrict the movement of suspended cocoa particles [24]. Previous studies in dairy beverages have shown that gellan can generate a structured or fluid-gel-like continuous phase capable of stabilizing colloidal and suspended particles, although the formation of a true yield-stress or weak-gel structure requires appropriate rheological characterization [25,26]. Colloidal stability in protein-based beverages is a complex phenomenon governed by the interplay of intermolecular interactions, particle organization, surface charge, phase behavior, and rheological properties rather than by a single physicochemical parameter. In mixed-protein systems, changes in pH and ionic strength can modify electrostatic interactions and protein–protein association, thereby affecting phase separation, aggregation, and particle organization. Previous studies have demonstrated that interactions between whey proteins and gellan gum can substantially affect the structural and physicochemical properties of protein-based systems. Gellan gum may interact with proteins (milk, soya bean, and egg), modify the surface-charge environment, enhance water immobilization, and increase the viscosity of the continuous phase. These effects can collectively influence particle organization and reduce particle movement, thereby hence gel formation [25]. In particular, FTIR analysis of whey protein isolate–high-acyl gellan gum systems demonstrated that pH significantly affected the interactions between WPI and gellan gum, highlighting the importance of the molecular environment in determining protein–polysaccharide interactions [26]. Similarly, Zand-Rajabi and Madadlou [27] reported interactions between whey proteins and gellan gum and demonstrated that gellan incorporation influenced the protein network structure and molecular interactions. These findings support the possibility that gellan gum can modify both molecular interactions and the organization of protein-based colloidal structures. In addition, the study by Salama et al. [28] demonstrates that colloidal behavior in mixed protein systems (milk proteins and egg white proteins) is governed by the interplay of intermolecular interactions, electrostatic forces, phase separation, particle organization, and environmental conditions.

Although gellan gum has clear potential as a stabilizer for milk-based cocoa beverages, its effectiveness depends on both formulation and processing conditions. In particular, the combined influence of gellan gum concentration and homogenization pressure on beverage stability, particle characteristics, microstructure, long-term storage behavior, and protein–polysaccharide interactions remain poorly understood. Therefore, this study aimed to evaluate gellan gum as a stabilizer in milk-based cocoa beverages and to optimize its performance by varying gellan gum concentration and homogenization pressure. We compared the optimized formulation with a commercial cocoa milk product using physicochemical, structural, sensory, and storage-stability analyses. Molecular docking simulations were also performed to investigate potential interactions between gellan gum and major milk proteins under simplified conditions that did not include other beverage components. Overall, this work was designed to provide practical and mechanistic insight into reducing sedimentation, creaming, and related instability defects in cocoa-based dairy beverages.

## 2. Materials and Methods

### 2.1. Materials

Pasteurized milk (3% fat, 3% protein, SEK (Süt endüstrisi kurumu anonim sirketi, Istanbul, Türkiye) and crystal sugar (Migros, Istanbul, Türkiye) were obtained from a local market in Istanbul, Türkiye. High acyl gellan gum powder (Kelcogel^®^ HM-Pure gellan gum) was kindly donated by CP Kelco (San Diego, CA, USA). Gellan gum’s main properties are as follows: molecular weight: 1000–2000 kDa, particle size: 355 µm, minerals (Ca, P, Na, K, respectively): 224 mg, 185 mg, 743 mg, 1660 mg. These properties were provided by the supplier. Alkalized cocoa powder was supplied by Altınmarka Cocoa and Chocolate Factory (Istanbul, Türkiye), potassium phosphate was supplied by Chemische Fabrik Budenheim KG (Rhineland-Palatinate, Germany), and potassium sorbate was supplied by CAC Nantong Chemical Co., Ltd. (Shanghai, China). In addition, a cocoa milk sample containing gellan gum was obtained from the local market as a control sample to compare the cocoa milk samples at the optimum point. All chemicals (potassium phosphate and potassium sorbate) used throughout the study were purchased from Sigma-Aldrich Chemie (Steinheim, Germany).

### 2.2. Sample Preparation and Experimental Design

Sugar (6%), alkalized cocoa powder (1%), potassium phosphate (0.50%), potassium sorbate (0.05%), and gellan gum were mixed in dry form using a laboratory balance in a glass container. The dry phase was added to the mixed pasteurized milk and all components were mixed at 500 rpm in Thermomix TM6 (Thermomix TM6, Vorwerk, Wuppertal, Germany). Changes in gellan gum addition rates were regulated by the amount of milk. The system was heated to 75 °C and homogenized at the pressure norms (between 58 and 341 bar) specified in the CCRD experimental design (Hommak C-HM1, İzmir, Türkiye) at the specified pressure. Pressure levels were determined by considering the pressure norms applied in the dairy industry. After homogenization, the sample was kept in Thermomix TM6 at 95 °C/4 min. The system was cooled very quickly to 25 °C and the sample was filled into glass vials with plastic caps.

The experimental design matrix and all data analyses were performed using Design-Expert^®^ (version 7.0, Stat-Ease Inc., Minneapolis, MN, USA). Response Surface Methodology (RSM) Central Composite Rotatable Design (CCRD) option was used to examine the effects of gellan gum concentration and homogenization pressure, two independent variables, for cocoa milk properties. The CCRD was chosen due to its ability to efficiently explore the effects of multiple variables and their interactions while minimizing the number of experiments required. This design is well-suited for optimization studies where both linear and quadratic effects need to be evaluated. Experimental design consists of 11 experimental steps and the effects of independent variables on WHC, sedimentation, creaming, pH, and viscosity values were investigated. The level range of gellan gum concentration (0.01–0.046%) and homogenization pressure (59–341 Bar) was determined using CCRD experimental design depending on the industrial applications. The selected range of gellan gum concentration and homogenization pressure was determined by preliminary experiments. All the responses were evaluated in terms of statistical significance of factors and *R*^2^ values. For each response variable, a second-order polynomial model equation was defined. Finally, validation studies were carried out under the optimum conditions obtained and L*, a*, b* colour values, zeta potential value, particle size, FTIR and microscope images were measured in the samples under these conditions. Optimization was conducted by minimizing sedimentation and creaming and maximizing WHC considering desirability value.

### 2.3. pH

Prior to the analysis of the samples, the pH meter was calibrated using standard buffers of pH 4.0 and 7.0 at 25 °C. The pH values of the end products (milk-based cocoa samples) were measured at 25 °C one day after production and subsequently at monthly intervals throughout the 9-month storage period with a pH meter (Mettler Toledo, Seven Compact, Giessen, Germany). All measurements were performed at least in triplicate and the results are presented as mean ± standard deviation.

### 2.4. Apparent Viscosity

Viscosity measurements were carried out with a viscometer (Lamy B-One Plus, Champagne, France) at a controlled temperature of 25 °C with a chosen speed of 100 rpm. Measurements were taken 2 min after the spindle was immersed in each sample, and to allow thermal equilibrium in the samples. Each measurement was triplicated on the same sample. Torque values were calculated as

Torque = Force × r × sin (θ).(1)

Whereas r (m) and sin (θ) were known as distance (lever arm (m)). Specifically, torque represents the rotational moment generated by the applied force about the axis of the mixing system.

### 2.5. Water Holding Capacity (WHC)

The water holding capacity of cocoa milk was determined by using a centrifugation (Nuve, NF 400, Ankara, Türkiye) of 50 mL sample at 4000 rpm for 10 min at room temperature. The released serum was measured as a volume (mL) [29]. WHC values were determined using the following formulation.

WHC%= [1 − ((Released serum (supernatant))/(original gel))] × 100(2)

### 2.6. Creaming Analysis

Prepared cocoa milk samples were transferred to glass bottle closed with plastic cap and stored at 4 ± 1 °C for 21 days. During this time, the emulsion samples were separated into two layers. These layers were distinguished by visual appearance. The creaming index (CI) of emulsions is calculated by the following equation:

(3) $$\mathrm{CI}\%\;=\;(H_{C}/H_{T})\times 100$$

where H_C_ (cm) is the height of the cream layer (top) and H_T_ (cm) is the total height of the milk sample [30].

### 2.7. Sedimentation

Prepared cocoa milk samples taken into glass bottle closed with plastic cap were stored at 4 ± 1 °C for 21 days. The sedimentation value (%) was expressed as the ratio of the height of the sediment at the bottom to the height of the milk sample [31].

(4) $$\mathrm{Sed}\%\;=(H_{S}/H_{T})\times 100$$

where H_S_ (cm) is the height of the sediment and H_T_ (cm) is the total height of the milk sample.

### 2.8. Color

The color was determined with a digital colorimeter (Konica Minolta, Chroma meter CR-410, Tokyo, Japan). L* indicates the lightness, a* and –a*, redness and greenness, b* and –b*, yellowness and blueness, respectively, according to the CIE L*, a*, b* colour space. At least three measurements were made for each sample and colour values were calculated as were given as mean ± standard deviation.

### 2.9. Fourier Transform Infrared (FTIR) Spectroscopy

FTIR spectra of milk-based cocoa beverages were recorded by a FTIR spectrophotometer (Thermo Nicolet, AVATAR-370 FTIR, Madison, WI, USA) at 4000–400 cm^−1^ wavenumber range [32]. The FTIR spectrophotometer was calibrated using standard reference materials prior to each measurement to ensure accuracy and reliability of spectra collected.

### 2.10. ζ-Potential and Droplet Size Measurements

The sample was diluted a hundredfold with phosphate buffer (5 mM). ζ-potential was measured using mastersizer (Nano ZS90, Malvern instrument Co., Ltd., Worcestershire, UK) at 25 °C, and measurements were performed triplicate. Droplet size distribution was measured by a Mastersizer 2000 (Malvern instrument Co., Ltd., Worcestershire, UK). Each 1 mL emulsion was diluted one thousand times with distilled water. The refractive indices were set at 1.33 for the continuous phase and 1.46 for the dispersed phase. The mean diameter value (d_4,3_) was calculated by:

(5) $$d_{4,3}\;=\;n_{i}{d_{i}}^{4}/\sum n_{i}{d_{i}}^{3}$$

where ni is the number of droplets of diameter di of sample respectively. The measurements were performed after the milk-based cocoa beverages preparation and results were obtained from average of three readings [33].

### 2.11. Microscope Images

The microstructure of cocoa milk produced with gellan gum at the optimum point and the commercial product was examined using a microscope (Zeiss EVO^®^ LS 10, Jena, Germany), magnified 10 and 40 times under the microscope.

### 2.12. Scanning Electron Microscopy (SEM) and Energy Dispersive Spectroscopy Analysis (EDS)

The microstructures of cocoa milk samples produced with gellan gum at optimum point and without hydrocolloid for comparison, and supplied from the market sample were analyzed using SEM-EDS (JEOL JIB-4601 MultiBeam FIB-SEM system, Tokyo, Japan). The samples were frozen at −20 °C and freeze dried for 48 h in a vacuum freeze dryer (Liyolife, FD5CT, Manisa, Türkiye) Scanning electron microscopy (SEM) and energy dispersive spectroscopy analysis (EDS) was carried out to reveal the microscopic features and chemical mapping.

### 2.13. Molecular Docking

Gellan gum was docked against α-lactalbumin (α-LA), β-lactoglobulin (β-LG), αs1-casein (αs1-CN) and β-casein (β-CN). The structures of α-LA (PDB 6IP9, 1.85 Å) [34] and unliganded β-LG (PDB 3NPO, 2.20 Å) [35]) were taken from the RCSB Protein Data Bank (https://www.rcsb.org, accessed on 21 October 2025), and water, sulfate, glycerol and the La^3+^ ion of 6IP9 were removed [36]. Casein models were built with I-TASSER (Zhang Lab, https://zhanggroup.org/I-TASSER/, accessed on 21 October 2025) [37] from the UniProt precursor sequences P02662 and P02666 (https://www.uniprot.org, accessed on 21 October 2025) without phosphate groups. Casein residues are numbered from the precursor, including the 15-residue signal peptide, so Arg134 of αs1-CN and Arg217 of β-CN correspond to Arg119 and Arg202 of the mature proteins [38] (Farrell et al., 2004). Hydrogens and Gasteiger charges [39] (Gasteiger and Marsili, 1980) were added in PyRx version 0.8 (The Scripps Research Institute, La Jolla, CA, USA), giving deprotonated Asp and Glu, protonated Lys and Arg and neutral His. These are the states expected at the measured beverage pH of 6.85 (Table 1) [40].

Gellan was represented by a tetrasaccharide oligomer (degree of polymerization 4) corresponding to one repeating unit of the backbone, α-L-Rhap-(1 → 3)-β-D-Glcp-(1 → 4)-β-D-GlcpA-(1 → 4)-β-D-Glcp [21] (O’Neill et al., 1983), with free hydroxyl groups at both termini. This is the smallest fragment that contains all four sugar residues and the single carboxyl group of the repeating unit, and it keeps the number of rotatable bonds manageable for the docking search. The structure was drawn in ChemDraw Professional 22.0 (PerkinElmer Informatics, Waltham, MA, USA) and converted to three-dimensional coordinates in PyRx. The neutral L-glyceryl and acetyl esters of high-acyl gellan [41] (Chandrasekaran et al., 1992) were omitted, and the glucuronic acid carboxyl, which is fully dissociated at pH 6.85, was modeled as a carboxylate with a net charge of −1. Cremer–Pople analysis [42] (Cremer and Pople, 1975) confirmed chair conformations for all four pyranose rings, and glycosidic and hydroxyl torsions were left free.

Docking was performed with AutoDock Vina 1.1.2 (The Scripps Research Institute, La Jolla, CA, USA) in PyRx 0.8. No carbohydrate binding site is known for these proteins, so the grid box enclosed the whole receptor. Box centers, given as x, y and z coordinates in Å, were (20.712, −23.973, −1.049) for α-LA, (−11.184, 11.717, 6.840) for β-LG, (8.087, −2.894, −0.446) for αs1-CN and (127.315, 120.036, 178.964) for β-CN. Each run used an exhaustiveness of 8 and returned nine binding modes within 3 kcal/mol, and the lowest-energy mode was analyzed. Interactions were identified with PLIP (Biotechnology Center, Technische Universität Dresden, Dresden, Germany, https://plip-tool.biotec.tu-dresden.de, accessed on 26 July 2026) [43], with salt bridges accepted only when they involved the glucuronate carboxylate, and were visualized with PyMOL version 2.5.5 (Schrödinger, LLC, New York, NY, USA), Discovery Studio Visualizer 2021 version v21.1 (Dassault Systèmes BIOVIA, San Diego, CA, USA), UCSF Chimera version 1.17.3 (Resource for Biocomputing, Visualization, and Informatics, University of California, San Francisco, CA, USA) and ProteinsPlus (Center for Bioinformatics, Universität Hamburg, Hamburg, Germany, https://proteins.plus, accessed on 26 July 2026). Other beverage components were not considered. Each docking was repeated with two additional random seeds. Because the caseins are intrinsically disordered [44], gellan was also docked against the four lower-ranked I-TASSER models of each casein.

### 2.14. Sensory Evaluation

The cocoa milk produced at the optimum point and cocoa milk containing gellan gum from the local market were kept at 4 ± 1 °C for 24. Sensory evaluation was carried out by a panel of 10 panelists, aged 20 to 32 years (4 men, 6 women), selected from master’s degree students taking a “sensory analysis” course at Yildiz Technical University and experienced in the sensory assessment process. A 5-point hedonic scale test was used for the sensory analysis, which consisted of smoothness, mouth feeling (texture and creaminess), homogeneity, flavor, and overall acceptability (1 = very bad, 2 = bad, 3 = middle, 4 = good, and 5 = excellent) [24].

### 2.15. Quality Properties During Storage

The cocoa milk produced at the optimum point and cocoa milk containing gellan gum from the local market’s storage analysis was carried out monthly for a 9-month period. In monthly periods, WHC, sedimentation, creaming, pH, viscosity, and Torque values were measured according to the methods mentioned above.

### 2.16. Statistical Analysis

All measurements taken have been repeated at least three times. The results were given as average value ± standard deviation. The significance of the independent factor was determined using ANOVA test. The differences among the samples were determined using the Tukey test. ANOVA was used to determine the statistical significance of the factors on the response variables. Tukey’s test was applied for pairwise comparisons among the experimental conditions. All these analyses were conducted using the IBM SPSS Statistics 14 (IBM Corp., Armonk, NY, USA) program at a significance level of 0.05.

## 3. Results and Discussion

**Figure 1:**
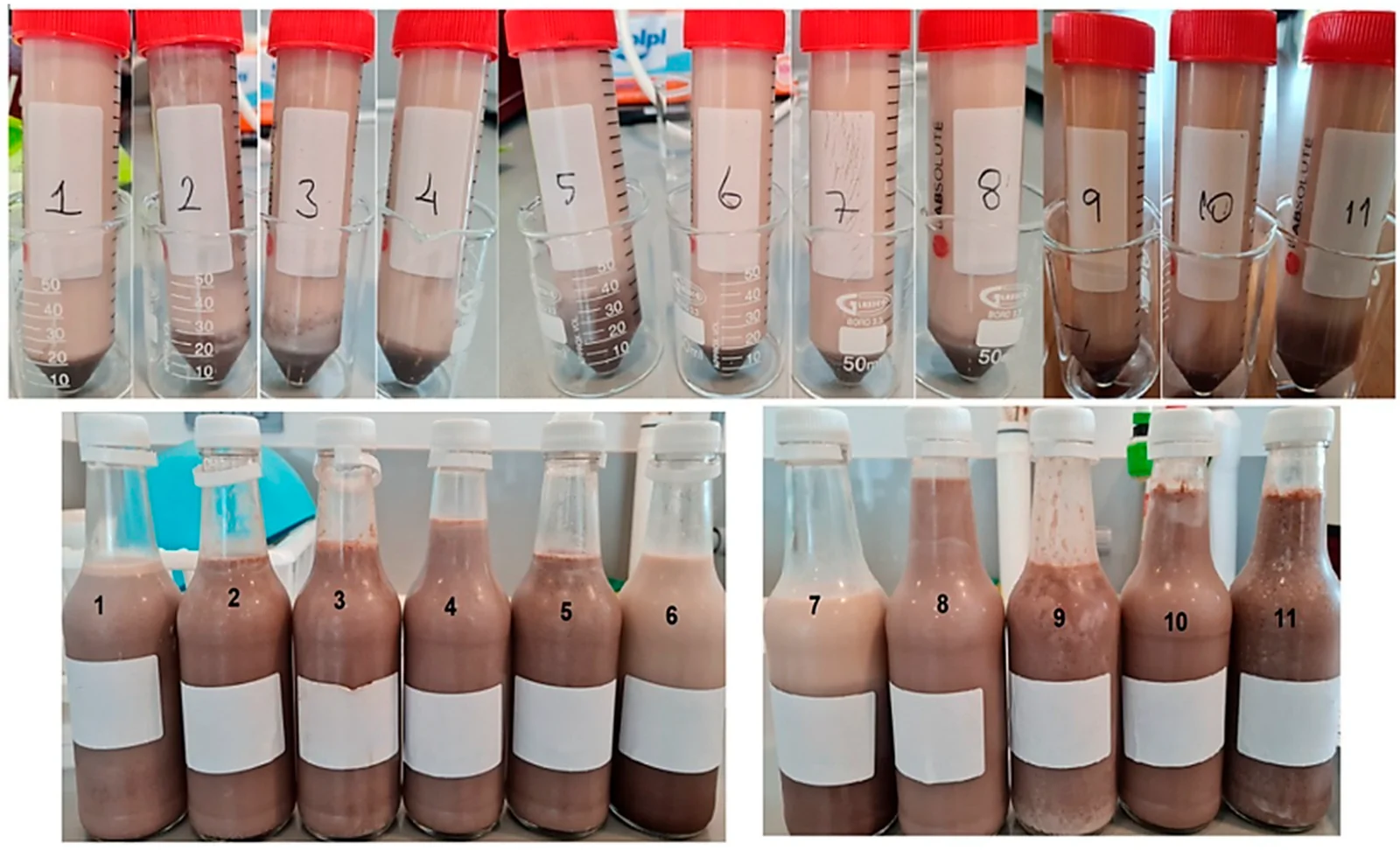
Images of milk-based cocoa beverages produced according to the CCD experimental design. The numbers on the cocoa milk bottles correspond to the experimental run numbers listed in Table 1 under the respective experimental conditions.

The physicochemical properties of the milk-based cocoa beverages produced under the different experimental conditions are summarized in Table 1, while their visual appearances are presented in Figure 1. Variations were observed among the formulations, particularly in water-holding capacity, sedimentation, creaming, viscosity, and torque, indicating that gellan gum concentration and homogenization pressure influenced the physical stability of the beverage system.

### 3.1. Quality Properties of Cocoa-Based Milk Beverages

#### 3.1.1. pH Measurement Results

**Figure 2:**
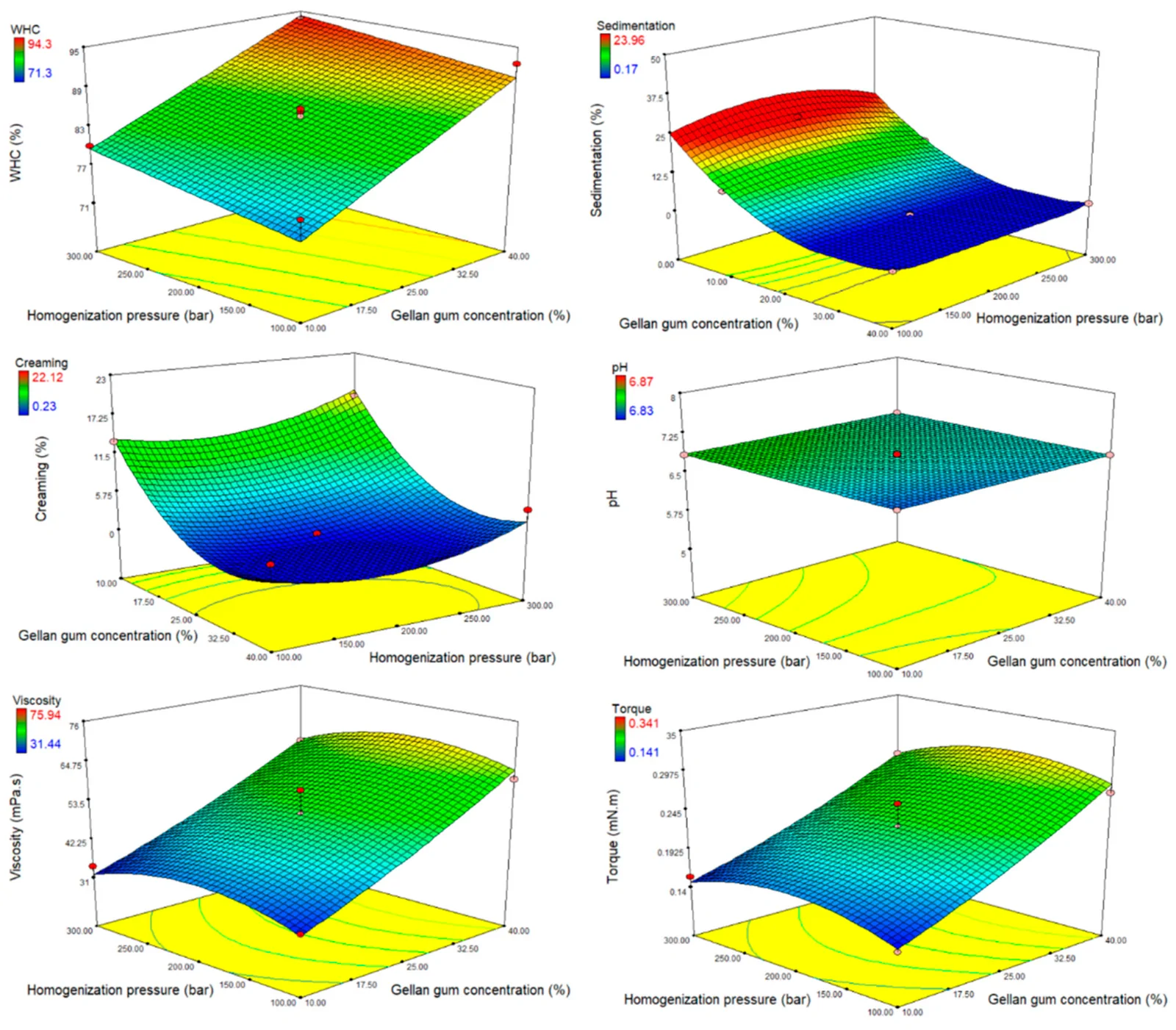
Effects of gellan gum concentration and homogenization pressure parameters on dependent parameters: From the top, from left to right WHC %, sedimentation (%), creaming (%), pH, viscosity (%) and Torque (mN·m) of the samples.

According to the CCRD results, neither gellan gum concentration nor homogenization pressure significantly affected the pH of the milk-based cocoa beverages (Figure 2). The relatively stable pH values observed across the experimental conditions indicate that the selected formulation and processing variables did not substantially alter the acid–base balance of the system.

Maintaining pH within a narrow range is important in dairy beverages because pH influences the net charge, solubility, and interaction behavior of milk proteins. As the pH approaches the isoelectric point of casein, reduced electrostatic repulsion may promote protein aggregation, whereas conditions that maintain sufficient repulsive forces can help preserve colloidal stability. Ionic strength also affects protein behavior interactions by modifying electrostatic screening, although excessive salt addition may adversely affect sensory quality. Therefore, processing approaches that improve milk-protein stability while maintaining desirable sensory properties are important in beverage development [45]. In the present study, the absence of a significant pH response suggests that the stability improvements were primarily related to rheological and structural changes rather than to pH variation.

#### 3.1.2. Viscosity and Torque Value

The viscosity of the milk-based cocoa beverages ranged from 31.44 to 75.94 mPa·s. A quadratic model adequately described the effects of gellan gum concentration and homogenization pressure on viscosity. Gellan gum concentration had a significant effect on viscosity (*p* < 0.05), whereas the linear effect of homogenization pressure was not significant (*p* > 0.05). The high coefficient of determination (R^2^ = 0.9575) indicated strong agreement between the experimental and predicted values and confirmed the suitability of the fitted model.

As shown in Figure 2, increasing gellan gum concentration increased apparent viscosity. This behavior can be attributed to the hydration of gellan gum and the formation of a more structured continuous phase, which increases resistance to flow. The resulting increase in viscosity may restrict the movement of dispersed cocoa particles and fat droplets, thereby reducing their tendency to migrate under gravitational forces. Consequently, the higher viscosity generated by gellan gum contributed to improved suspension stability.

Although linear effect of homogenization pressure was not significant, its quadratic effect was significant (*p* < 0.05). This non-linear response suggests that pressure may influence viscosity indirectly by altering particle size, particle–particle interactions, and the organization of the dispersed phase. However, the precise mechanisms underlying this nonlinear behavior cannot be established from the response-surface results alone.

Torque values exhibited a trend similar to that of viscosity. Increasing gellan gum concentration significantly increased torque (*p* < 0.05), whereas homogenization pressure had no significant linear effect (*p* > 0.05). These findings indicate that gellan gum acts not only as a thickening agent but also as a structural stabilizer that increases resistance to flow and limits particle displacement. Similar effects of gellan gum on viscosity, microstructure, and stability have been reported in milk-based systems [46]. Overall, the observed increases in viscosity and torque suggest that gellan gum contributed to the development of a more structured beverage system, which may help limit particle migration and reduce phase separation.

#### 3.1.3. Water Holding Capacity (WHC) Results

The water-holding capacity (WHC) of the beverages ranged from 71.3% to 94.3%. A linear model adequately described the response, and both gellan gum concentration and homogenization pressure significantly affected WHC (*p* < 0.05). The coefficient of determination (R^2^ = 0.9346) demonstrated a satisfactory fit between the model and the experimental data.

As illustrated in Figure 2, WHC increased substantially with increasing gellan gum concentration. This effect is primarily due to the large number of hydrophilic groups in the gellan gum molecule, which promote water binding and reduce aqueous-phase mobility. Increasing gellan gum concentration likely generated a denser, more continuous polysaccharide network, improving water entrapment and reducing serum release.

Homogenization pressure also significantly affected WHC (*p* < 0.03), although its effect was less pronounced than that of gellan gum concentration (*p* < 0.001). The positive effect of homogenization pressure may be associated with improved dispersion of cocoa particles and reduced particle size, which increases the effective surface area available for interaction with the surrounding aqueous phase and hydrocolloid network. Similar improvements in WHC have been reported for hydrocolloid-containing dairy and gelled food systems [17,47,48].

From a practical perspective, higher WHC may help limit serum separation and maintain beverage homogeneous beverage throughout storage. Improved water retention also supports viscosity and structural integrity, thereby reducing the likelihood of creaming and sedimentation. In addition, a beverage with adequate WHC is more likely to maintain a smooth and consistent mouthfeel over its shelf life.

#### 3.1.4. Creaming

Creaming values ranged from 0.23% to 22.12% (Table 1). A quadratic model adequately described the response, with a R^2^ value of 0.9646. Gellan gum concentration significantly affected creaming (*p* < 0.05), whereas the linear effect of homogenization pressure was not significant (*p* > 0.05).

As shown in Figure 2, increasing gellan gum concentration markedly reduced creaming. This effect can be explained by the formation of a weak gel-like network within the continuous phase, which increases viscosity and restricts the upward migration of fat droplets. Once the gellan gum concentration exceeded approximately 0.02%, the creaming index approached its minimum level, and further increases in concentration produced little additional benefit. This plateau suggests that a sufficiently developed network had already formed to immobilize the dispersed droplets.

Although the linear effect of homogenization pressure was not significant (*p* < 0.05), This response may reflect pressure-related changes in droplet size distribution and interactions among fat droplets, milk proteins, and other dispersed components. In milk systems without hydrocolloids, pressure treatments between 100 and 250 MPa have been reported to increase creaming, whereas treatments at 400–600 MPa reduced creaming by up to 70% [49]. However, this pressure conditions applied in the present study was substantially lower and was used primarily for homogenization rather than high-pressure processing. The observed reduction in creaming therefore appears to be driven mainly by increased continuous-phase viscosity and the formation of a gellan gum–mediated network, consistent with previous findings on hydrocolloid-stabilized milk systems [30].

#### 3.1.5. Sedimentation Results

Sedimentation values ranged from 0.17% to 23.96%. A quadratic model adequately described the relationship between the processing variables and sedimentation, with an R^2^ value of 0.9546. Gellan gum concentration significantly affected sedimentation (*p* < 0.05), whereas homogenization pressure did not exert a significant linear effect (*p* > 0.05).

As shown in Figure 2 and Table 1, increasing gellan gum concentration substantially reduced sedimentation. The response reached a minimum when the gellan gum concentration exceeded approximately 0.02%, after which further increases produced only limited additional improvement. This behavior is consistent with the formation of a sufficiently strong weak-gel network capable of suspending insoluble cocoa particles.

Previous studies have similarly shown that gellan gum reduces sedimentation in beverage systems by increasing water retention and forming a continuous structural network [5,24,31,46]. In the present study, gellan gum likely reduced the mobility of cocoa particles by increasing the viscosity of the serum phase and generating a weak yield-stress structure. These effects decrease the rate of particle settling and limit aggregation. Rather than surrounding each particle individually, gellan gum more plausibly stabilizes the suspension by modifying the rheology of the continuous phase and contributing to a network that restricts particle movement. The combined reductions in sedimentation and creaming indicate that gellan gum effectively improved both the suspension and emulsion stability of the milk-based cocoa beverage.

### 3.2. Optimization and Comparison with Local Market Product

A numerical optimization was performed using the CCRD model. The optimization objective was to maximize WHC, minimize sedimentation and creaming, and maintain pH, viscosity, and torque within desirable ranges. The optimal conditions were identified as 0.04% gellan gum and a homogenization pressure of 214.82 bar. Under these conditions, the model predicted a WHC of 92.67%, creaming of 0.311%, pH of 6.84, viscosity of 66.13 mPa·s, torque of 0.297 mN·m, and no detectable sedimentation. The overall desirability value was 0.975, indicating that the selected formulation provided an effective compromise among the multiple response variables. The experimentally measured values were in close agreement with the predicted values, confirming the predictive reliability of the optimization model (Table 2).

The optimized beverage was subsequently compared with a commercially available milk-based cocoa beverage. No sedimentation was detected in the optimized formulation, whereas the commercial sample showed measurable sedimentation. The commercial beverage also exhibited a higher creaming value, indicating greater phase separation. In addition, the commercial product had a higher pH, viscosity, and torque than the optimized beverage. The higher pH may reflect the use of alkalized cocoa, buffering salts, or other formulation differences, although this interpretation cannot be confirmed solely from the present data. Importantly, the optimized product achieved better physical stability without requiring the higher viscosity observed in the commercial sample, suggesting that stabilization was not dependent exclusively on thickening. The optimized cocoa milk exhibited physicochemical and stability characteristics that differed from those of the commercial product. However, the commercial product was evaluated only as a market reference, and its exact formulation, cocoa type, stabilizer composition and concentration, heat-treatment conditions, and homogenization parameters were not available. Therefore, the observed differences should be interpreted as product-specific differences rather than evidence of overall superiority of the optimized formulation.

Color, particle size, zeta potential, FTIR, microscopy, and SEM-EDS analyses were used to further compare the optimized and commercial formulations. The commercial product exhibited a lower L* value, indicating a darker appearance (Table 3). This difference may be related to the use of chocolate powder, the degree of cocoa alkalization, cocoa variety, origin, or processing history.

The optimized beverage had a substantially smaller mean particle size than the commercial product. Because sedimentation velocity increases with particle diameter, the reduction in particle size likely contributed directly to the improved physical stability of the optimized formulation. Smaller particles remain suspended more readily and are less susceptible to gravitational separation, particularly when combined with the increased viscosity and weak-gel structure provided by gellan gum.

The optimized sample also exhibited a zeta potential of −38.73 mV, compared with −27.00 mV for the commercial product. The higher absolute zeta potential indicates stronger electrostatic repulsion among dispersed particles and a reduced tendency toward aggregation. Although no universal threshold defines colloidal stability for all food systems, values approaching or exceeding an absolute magnitude of 30 mV are commonly associated with improved electrostatic stabilization. The more negative zeta potential of the optimized beverage therefore supports its enhanced resistance to particle aggregation and phase separation. Hydrocolloids can improve colloidal stability by modifying the interfacial environment and creating steric or electrostatic barriers around dispersed particles [5,50].

Comparable zeta potential values have been reported for milk systems stabilized with carboxymethyl cellulose and soybean soluble polysaccharides [51]. The combination of reduced particle size and increased absolute zeta potential observed in the optimized formulation likely contributed to the reduction in both sedimentation and creaming. These structural improvements are also consistent with the smoother and more homogeneous sensory and microscopic characteristics of the optimized beverage.

**Figure 3:**
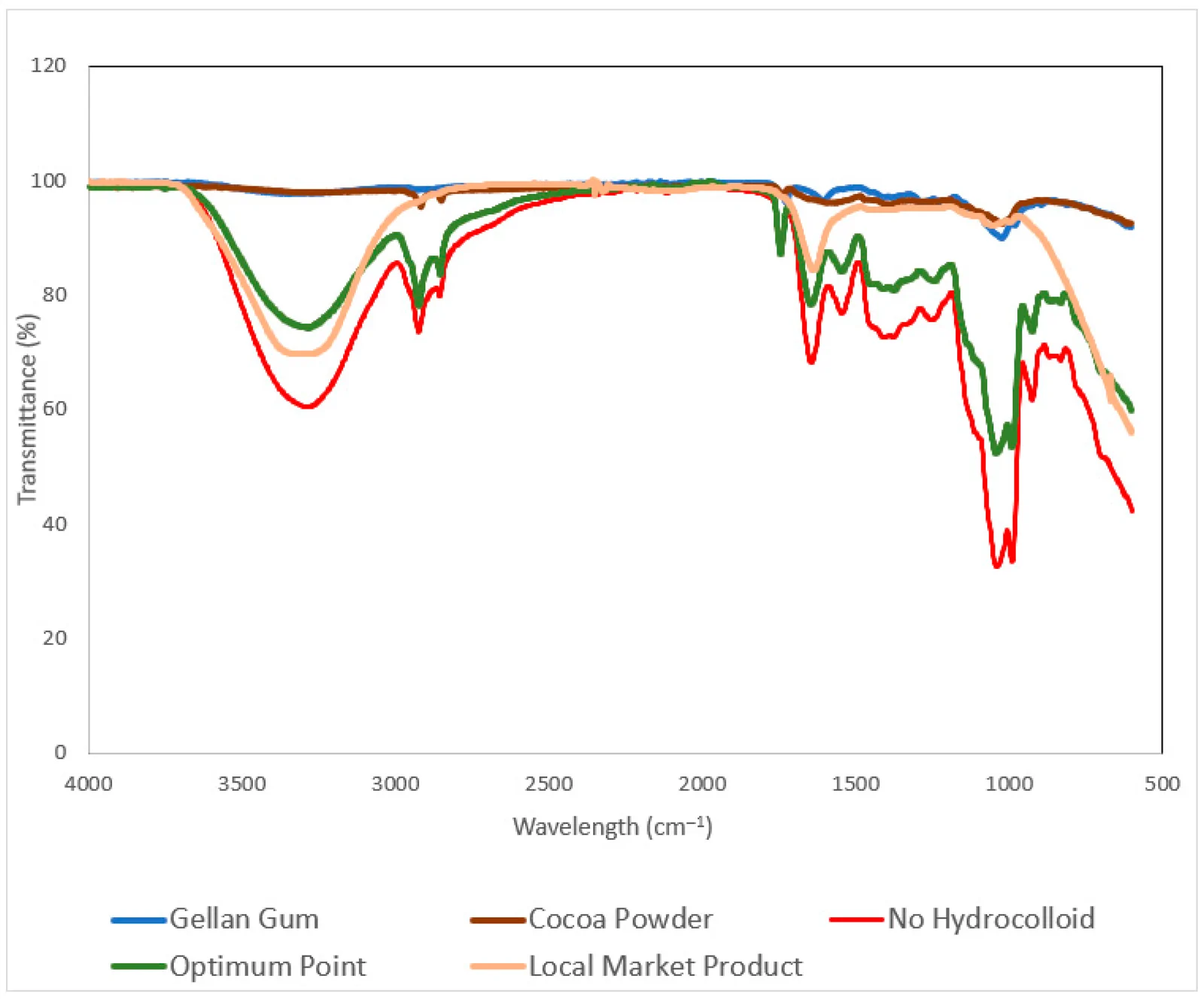
FTIR Analysis results of optimum sample and local market product and gellan gum and cocoa powder.

FTIR analysis was conducted to assess the major functional groups and possible molecular interactions within the beverage system (Figure 3). The broad absorption band between 3100 and 3600 cm^−1^ was assigned to O–H stretching vibrations associated with water, polysaccharides, and other hydroxyl-containing components. The intensity of this region is consistent with the hydrophilic nature of gellan gum and its contribution to water binding. Bands in the 1550–1650 cm^−1^ region were associated with protein-related amide vibrations and carbonyl-containing groups, whereas peaks between 1010 and 1080 cm^−1^ were attributed mainly to C–O and C–C stretching vibrations in polysaccharides and glycosidic linkages. Changes in the spectral profiles of the optimized cocoa milk compared with the control were observed, suggesting alterations in the molecular environment of the functional groups, which may be associated with hydrogen bonding and other molecular interactions following gellan incorporation. Therefore, in the revised manuscript, the observed changes are described conservatively as evidence of possible alterations in protein–polysaccharide interactions rather than as direct proof of a specific conformational change. This interpretation is also consistent with studies in which FTIR was combined with complementary techniques to confirm protein–gellan interactions [52]. FTIR analysis demonstrated that pH affected the interactions between whey protein and gellan gum, supporting the sensitivity of the protein–polysaccharide interactions to the molecular environment [53]. Zand-Rajabi and Madadlou [27] reported changes in the FTIR spectra of whey protein hydrogels containing gellan, with the results suggesting changes in protein secondary structure and interactions between the carboxyl groups of gellan and amino groups of β-lactoglobulin. More recently, FTIR analysis of pea protein–gellan gum systems showed a shift in the O–H/N–H band toward lower wavenumbers after gellan incorporation, which was attributed to hydrogen-bonding interactions; slight changes in the amide I, II, and III bands were also associated with rearrangement of the protein secondary structure [54].

These assignments are consistent with previous FTIR studies of hydrocolloid-containing food systems [28,55]. The optimized and commercial beverages exhibited broadly similar spectral patterns, indicating that both contained comparable major classes of food components. However, changes in peak intensity and shape may reflect differences in hydrogen bonding, hydration, and interactions among gellan gum, milk proteins, and cocoa-derived components. Because substantial peak shifts were not reported, the spectra should be interpreted as evidence of association and network formation rather than the creation of new covalent bonds. Overall, the FTIR results support the role of non-covalent interactions in the stabilization of the optimized beverage.

**Figure 4:**
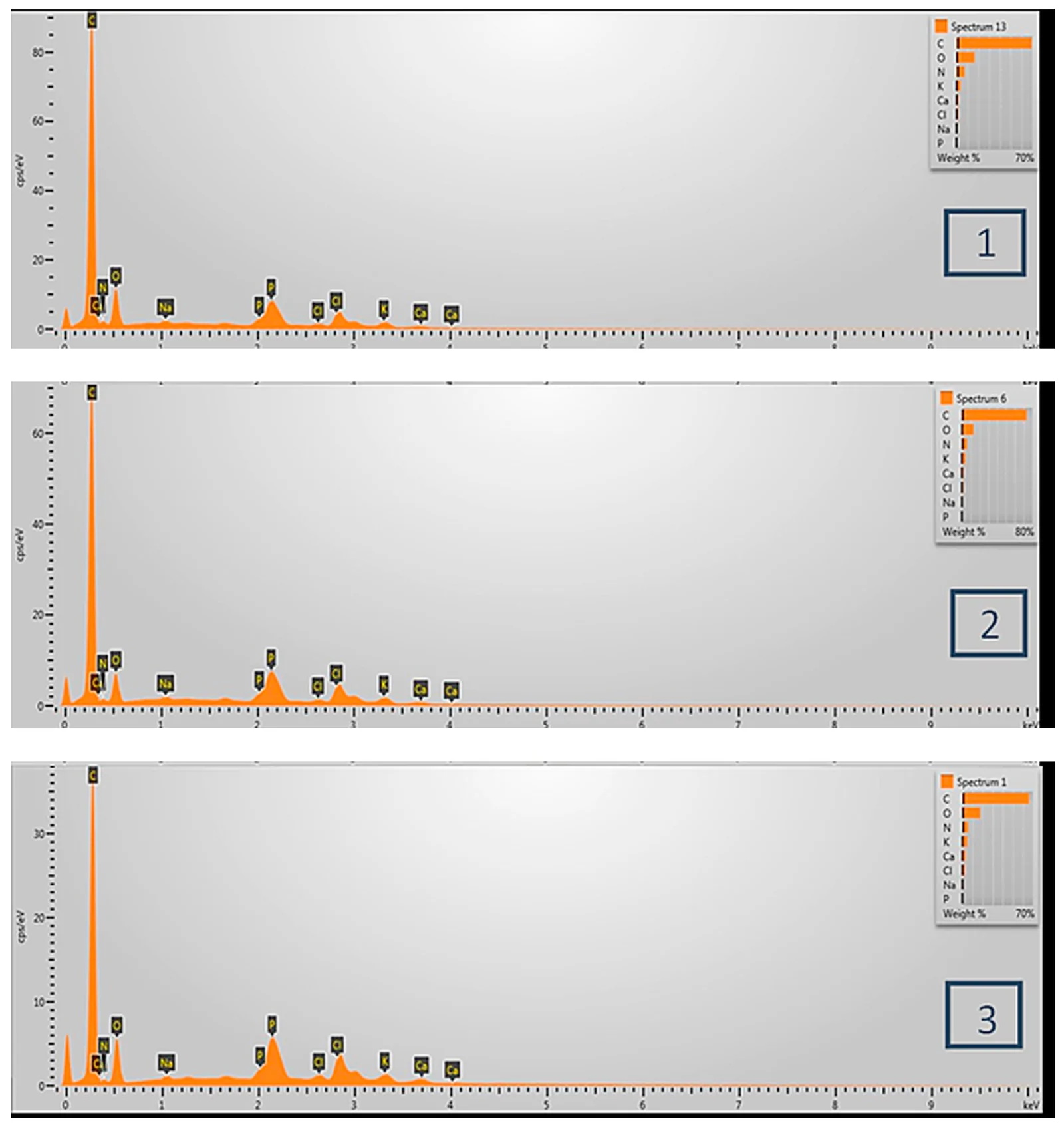
EDS images of cocoa milk samples without hydrocolloid (**1**), optimum sample (**2**) and commercial product (**3**), respectively (Elements written on the peaks are C, N, O, Na, P, P, Cl, Cl, K, Ca, Ca, respectively).

EDS analysis revealed differences in elemental composition among the samples (Table 4 and Figure 4). The commercial beverage contained higher levels of sodium, chlorine, and calcium than the optimized formulation. These differences may indicate the use of additional salts, mineral fortification, or formulation-specific ingredients; however, the precise source of these elements cannot be determined from EDS alone. The optimized beverage contained 0.48% sodium and 1.49% chlorine, whereas the commercial product contained 0.74% sodium and 1.99% chlorine. The more balanced elemental distribution observed in the optimized sample may reflect a more uniform matrix after drying and sample preparation (Table 4). Nevertheless, EDS data should be interpreted cautiously because the measured composition may also be influenced by sample preparation, local heterogeneity, and the semi-quantitative nature of the technique.

Overall, the physicochemical, microscopic, and structural analyses consistently demonstrated that the optimized combination of gellan gum concentration and homogenization pressure produced a more homogeneous and physically stable milk-based cocoa beverage than either the hydrocolloid-free or commercial formulations.

**Figure 5:**
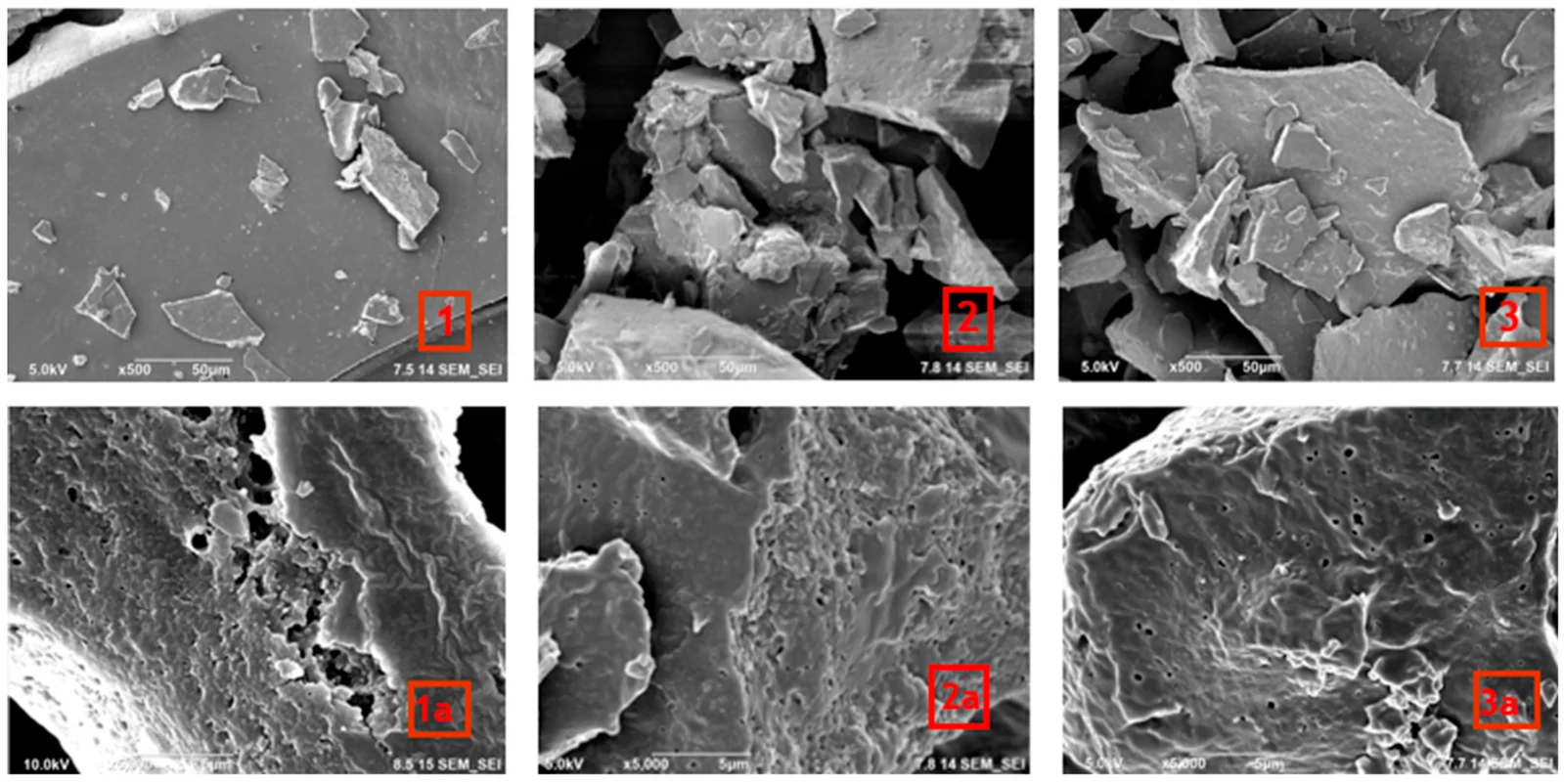
SEM images of samples without hydrocolloid (**1**,**1a**), optimum cocoa milk (**2**,**2b**) and commercial product (**3**,**3b**) with 500-magnification and 5000-magnifications (**1a**,**2a**,**3a**).

Microscopic observations revealed clear structural differences among the optimized, commercial, and hydrocolloid-free samples (Figure 5). The optimized formulation showed a more uniform and continuous microstructure, with fewer visible regions of serum separation. This morphology is consistent with improved particle dispersion and a more cohesive continuous phase. In contrast, the hydrocolloid-free sample exhibited greater structural discontinuity and visible signs of instability.

**Figure 6:**
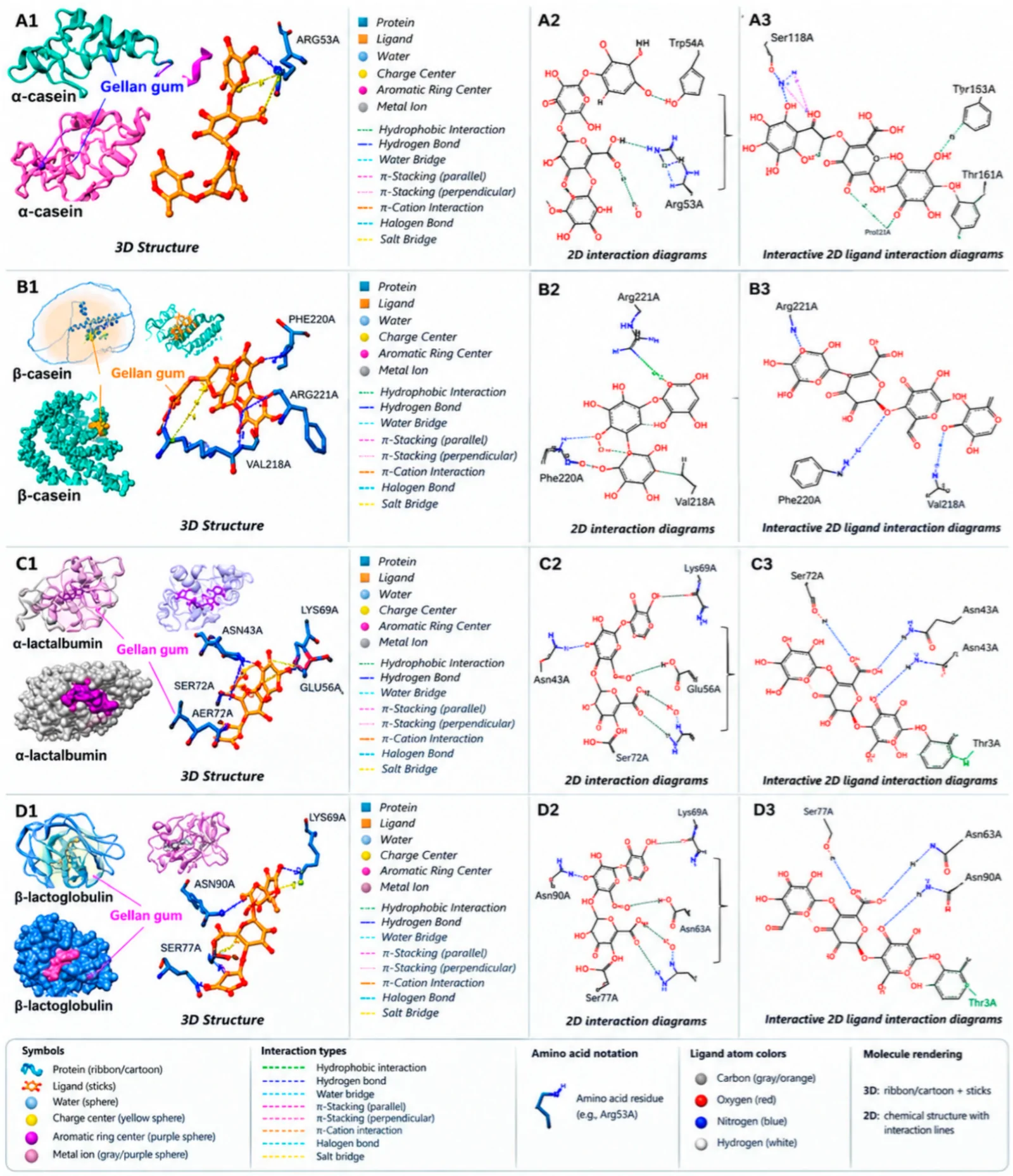
Molecular interactions between gellan gum and milk proteins: α-casein (**A**), β-casein (**B**), α-lactalbumin (**C**), and β-lactoglobulin (**D**). The 3D structures (**A1**–**D1**) illustrate the predicted binding configurations of gellan gum with the respective proteins, while the 2D interaction diagrams (**A2**–**D2**) and interactive 2D ligand interaction diagrams (**A3**–**D3**) provide detailed representations of the intermolecular interactions and the amino acid residues involved.

SEM observations further supported these findings in Figure 6. The hydrocolloid-free sample displayed numerous voids and a sponge-like structure, indicating weak cohesion and poor network formation. The optimized sample showed fewer voids and a smoother, denser, and more integrated microstructure, suggesting the formation of a continuous hydrocolloid–protein matrix. The commercial sample exhibited a more cohesive structure than the hydrocolloid-free control but was less uniform than the optimized formulation. These results are consistent with reports that gellan gum and other hydrocolloids can improve microstructural continuity and stability in complex food matrices [55,56].

### 3.3. Molecular Docking for the Optimum Sample

Molecular docking was used to explore potential interactions between gellan gum and major bovine milk proteins and to provide a mechanistic interpretation of the experimentally observed stability (Figure 6). The simulations were conducted under simplified conditions in which the possible effects of lactose, cocoa polyphenols, salts, and other beverage components were excluded. The predicted binding affinities were protein dependent. Among the proteins examined, α-lactalbumin exhibited the strongest predicted binding affinity, with a binding energy of −7.8 kcal/mol, followed by β-lactoglobulin at −7.3 kcal/mol. Lower affinities were predicted for αs1-casein and β-casein, with binding energies of −5.5 and −5.1 kcal/mol, respectively.

The higher predicted affinities for α-lactalbumin and β-lactoglobulin may be related to their compact globular structures and the presence of more defined binding pockets. Hydrogen bonding, hydrophobic interactions, and salt bridges contributed to the stabilization of the predicted complexes. In the α-lactalbumin complex, six hydrogen bonds involving Asn44, Ser47, Glu49, Asn56, Leu105, and Ala106 were identified, together with hydrophobic interactions involving residues such as Tyr103. For β-lactoglobulin, four hydrogen bonds were predicted, primarily involving Lys69 and Asn88. Lys69 also participated in a salt bridge with gellan gum, which may strengthen electrostatic attraction between the polysaccharide and protein.

The predicted interactions with caseins were weaker but still included hydrogen bonds and electrostatic contacts. In αs1-casein, Arg134 contributed to hydrogen bonding and salt-bridge formation, whereas β-casein formed four hydrogen bonds and a salt bridge involving Arg217. At the beverage pH of 6.85 both gellan and the caseins carry a net negative charge, so these contacts are better read as local association of the glucuronate carboxyl with exposed arginine side chains, the patch-type interaction expected for an anionic polysaccharide above the isoelectric point of a protein, than as binding to a defined site. These findings suggest that gellan gum can interact with both whey proteins and caseins, although the scores are more favorable for the globular whey proteins.

The docking results are consistent with the physical stability improvements observed experimentally. The predicted hydrogen bonding, hydrophobic interactions, and salt bridges between gellan gum and milk proteins may contribute to the formation of a more interconnected matrix that limits aggregation and phase separation [56]. The stronger predicted affinities for α-lactalbumin and β-lactoglobulin compared with caseins are in agreement with previous evidence that hydrogen bonding, hydrophobic interactions, and electrostatic forces contribute to protein–ligand association [36].

Nevertheless, the docking results should be interpreted as supportive rather than definitive evidence because the beverage is a multicomponent system and the simulations did not account for competing interactions with cocoa compounds, lactose, minerals, or other macromolecules. Within this limitation, the molecular modeling provides a plausible mechanism linking gellan gum–protein interactions with the observed increase in absolute zeta potential and reduction in sedimentation and creaming.

The gelation behavior of gellan gum is also strongly influenced by cations, particularly Ca^2+^. The carboxylate groups of gellan gum can interact with divalent cations, creating junction zones between adjacent polymer chains and promoting the formation of a three-dimensional network [57]. The extent of this ionic cross-linking depends on gellan gum concentration, calcium availability, temperature, and other formulation conditions. Therefore, the stabilization mechanism likely involves both protein–polysaccharide interactions and cation-mediated cross-linking of gellan gum chains [12,57].

The predicted interactions between gellan gum and α-lactalbumin, including those involving Asn44, Ser47, and Glu49, may contribute to a more cohesive molecular network. Similarly, hydrophobic interactions in the β-lactoglobulin complex and the salt bridge involving Lys69 may support protein association with the gellan gum matrix. These molecular-level interactions are consistent with the high absolute zeta potential of −38.73 mV and the absence of sedimentation under the optimized conditions.

#### 3.3.1. Sensory Evaluation Results

**Figure 7:**
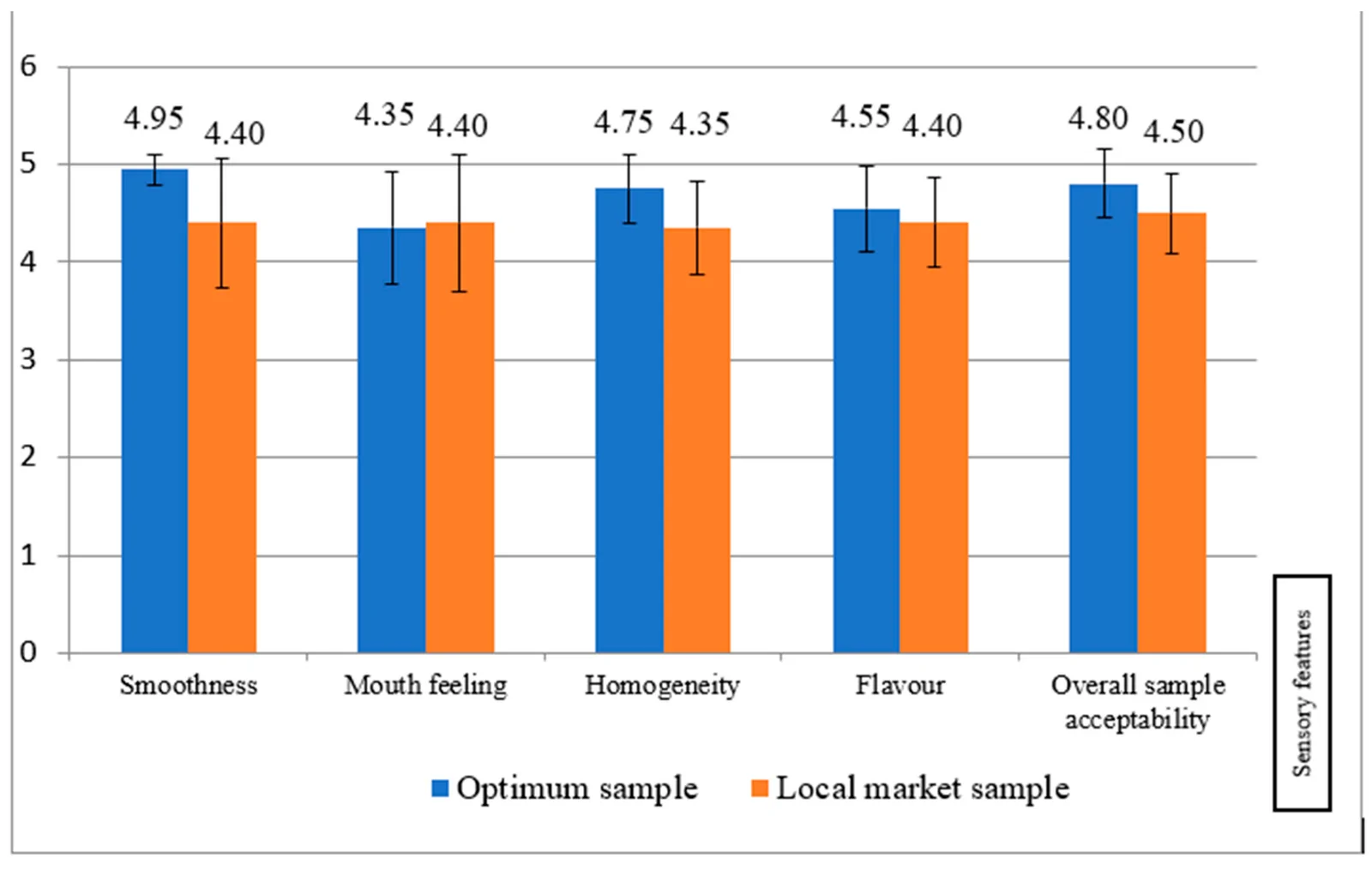
The sensory evaluation scores for optimum cocoa milk and commercial cocoa milk containing gellan gum.

The sensory properties of the optimized and commercial cocoa milk beverages were evaluated in terms of smoothness, mouthfeel, homogeneity, flavor, and overall acceptability (Figure 7). Both samples received high scores across all attributes, indicating good sensory quality. Smoothness is particularly important in cocoa milk because coarse or poorly dispersed cocoa particles can produce a gritty mouthfeel. Both beverages received smoothness scores above 4.30, suggesting that the panelists perceived minimal roughness. This observation is consistent with the particle-size and microstructural results, which indicated effective dispersion in both products.

Mouthfeel scores were also comparable between the optimized and commercial samples. The optimized formulation therefore achieved sufficient viscosity and creaminess without becoming excessively thick. Homogeneity scores were similarly high, indicating a uniform distribution of cocoa particles and other components throughout the beverage. These results support the effectiveness of the gellan gum network in maintaining a visually and texturally consistent system.

Both samples exhibited acceptable cocoa flavor and aroma, with no strong undesirable notes such as excessive bitterness, astringency, or sourness. The high overall acceptability scores indicate that the optimized formulation achieved sensory quality comparable to that of the commercial product. These findings are important because physical stabilization is only technologically useful when it does not compromise flavor or mouthfeel.

#### 3.3.2. Shelf Stability Analysis During Storage

The optimized and commercial cocoa milk beverages were monitored for nine months to evaluate changes in WHC, sedimentation, creaming, pH, viscosity, and torque. In the optimized sample, WHC decreased slightly over time; however, the change was not statistically significant (*p* < 0.05) (Table 5 and Table 6). In contrast, the commercial product exhibited a significant (*p* > 0.05) reduction in WHC during storage, indicating a progressive loss of water retention and structural integrity. Sedimentation also differed between the two products. No detectable sedimentation was observed in the optimized beverage throughout the storage period, whereas the commercial product showed a significant increase in sedimentation over time.

Creaming remained low and relatively stable in the optimized formulation but increased substantially in the commercial beverage. The stability of both sedimentation and creaming in the optimized product confirms the effectiveness of the weak gellan gum network in restricting the movement of cocoa particles and fat droplets.

The pH of both beverages remained relatively stable during storage, indicating no substantial change in their acid–base properties. Apparent viscosity increased in both samples, although the increase was much greater in the commercial product. In the optimized sample, apparent viscosity increased from approximately 59.50 to 71.23 mPa·s over nine months, whereas the commercial sample increased from 79.75 to 135.00 mPa·s. This pronounced increase in the commercial product may reflect progressive particle aggregation, restructuring of the dispersed phase, or continued hydration of stabilizers.

Torque followed a similar pattern. No statistically significant change was observed in the optimized formulation, whereas the commercial beverage showed a marked increase during storage. The relative stability of torque in the optimized product indicates that its internal structure remained comparatively consistent, whereas the increasing torque of the commercial beverage suggests progressive structural changes.

Overall, the optimized milk-based cocoa beverage exhibited superior long-term shelf stability compared with the commercial product. The combination of gellan gum and optimized homogenization pressure maintained WHC, prevented sedimentation, minimized creaming, and limited changes in rheological behavior. These effects are consistent with the formation of a stable hydrocolloid-mediated network that restricted particle aggregation and phase separation throughout refrigerated storage.

## 4. Conclusions

This study demonstrated that optimizing gellan gum concentration in combination with homogenization pressure can improve the physicochemical stability of milk-based cocoa beverages. Gellan gum increased water-holding capacity and apparent viscosity while reducing sedimentation and creaming, whereas homogenization contributed to improved particle dispersion and supported the development of a more stable colloidal system. Under the optimized conditions, the beverage exhibited high WHC, no detectable sedimentation, minimal creaming, smaller particle size, and a higher absolute zeta potential than the commercial product, indicating enhanced resistance to particle aggregation and phase separation.

The optimized formulation also maintained its stability during nine months of refrigerated storage. WHC, sedimentation, and creaming remained comparatively stable, while changes in viscosity and torque were more limited than those observed in the commercial beverage. The physicochemical findings were supported by FTIR, microscopic, SEM-EDS, and molecular docking analyses, which collectively suggested that gellan gum improved stability through water binding, network formation, protein–polysaccharide interactions, and electrostatic effects. Importantly, the optimized beverage achieved sensory properties comparable to those of the commercial product.

Overall, the results indicate that gellan gum combination with an appropriate homogenization pressure, is an effective strategy for improving homogeneous, physically stable, and sensorially acceptable milk-based cocoa beverages. Further studies should evaluate consumer acceptance, processing performance at pilot and commercial scales, cost-effectiveness, and shelf-life stability under a broader range of storage and distribution conditions.

## Figures and Tables

**Table 1:** The response values obtained with the run points of the created CCRD experimental design.

Run	Gellan Gum Concentration (%)	Homogenization Pressure (Bar)	WHC (%)	Sedimentation (%)	Creaming (%)	pH	Apparent Viscosity (mPa·s)	Torque (mN·m)
1	0.010	300	80.00 ± 2.83	10.00 ± 1.18	16.15 ± 1.09	6.84 ± 0.01	34.28 ± 2.14	0.154 ± 0.002
2	0.046	200	94.30 ± 2.40	0.33 ± 0.001	0.69 ± 0.11	6.84 ± 0.02	75.94 ± 1.97	0.341 ± 0.001
3	0.025	200	84.60 ± 0.57	0.40 ± 0.14	0.23 ± 0.09	6.84 ± 0.01	49.75 ± 0.13	0.223 ± 0.004
4	0.025	341.4	88.70 ± 0.99	0.94 ± 0.15	8.27 ± 0.82	6.87 ± 0.01	36.56 ± 0.72	0.164 ± 0.004
5	0.040	300	93.60 ± 0.85	0.75 ± 0.12	5.81 ± 0.49	6.83 ± 0.01	58.74 ± 0.63	0.264 ± 0.008
6	0.003	200	71.30 ± 0.99	23.96 ± 0.88	22.12 ± 1.36	6.85 ± 0.02	31.44 ± 0.78	0.150 ± 0.003
7	0.010	100	78.00 ± 2.83	10.88 ± 1.12	13.27 ± 0.82	6.83 ± 0.01	33.44 ± 1.28	0.141 ± 0.001
8	0.025	58.5	78.80 ± 1.70	0.94 ± 0.03	3.42 ± 0.60	6.84 ± 0.01	42.38 ± 1.31	0.190 ± 0.006
9	0.025	200	85.40 ± 0.57	0.21 ± 0.06	0.56 ± 0.08	6.84 ± 0.01	46.18 ± 1.41	0.207 ± 0.002
10	0.025	200	85.70 ± 0.99	0.33 ± 0.04	0.88 ± 0.05	6.85 ± 0.02	56.63 ± 1.69	0.254 ± 0.002
11	0.040	100	92.50 ± 0.71	0.78 ± 0.07	4.27 ± 0.60	6.84 ± 0.01	59.78 ± 1.11	0.269 ± 0.006
Model			Linear	Quadratic	Quadratic		Quadratic	
lack-of-fit (LOF)			16.45	31.33	49.61	4.50	1.65	0.25
*p* value			<0.0001 *	0.0048 *	0.0024 *		0.8196 **	
R^2^			0.9346	0.9536	0.9646		0.9575	
Adjusted-R^2^			0.9178	0.9072	0.9291		0.9151	
Predicted- R^2^			0.8510	0.6704	0.7499		0.8385	

*; significant **; not significant, Mean ± Standard deviation. All analysis performed at least in triplicate.

**Table 2:** Optimum point obtained by CCRD analysis results of optimum sample and local market product.

Products	WHC (%)	Sedimentation (%)	Creaming (%)	pH	Apparent Viscosity (mPa·s)	Torque (mN·m)
Optimum predicted	92.67	nd	0.311	6.84	66.13	0.297
Optimum experimental	93.00 ± 0.28 ^a^	nd ^b^	0.012 ± 0.005 ^b^	6.85 ± 0.01 ^a^	59.44 ± 0.50 ^b^	0.268 ± 0.002 ^a^
Local Market Product	86.45 ± 0.78 ^b^	3.72 ± 0.34 ^a^	2.95 ± 0.16 ^a^	7.37 ± 0.03 ^a^	76.20 ± 0.47 ^a^	0.342 ± 0.002 ^a^

nd: Not detected. The standard deviation is represented by ±values (*n* = 3). Different letters in each column (according to Tukey test) are statistically significantly different (*p* < 0.05).

**Table 3:** Analysis results of optimum sample and local market product.

Products	L*	a*	b*	Z-Ave (nm)	Zeta Potential (mV)
Optimum sample	52.17 ± 0.18 ^a^	8.17 ± 0.06 ^b^	8.86 ± 0.19 ^b^	309.90 ± 7.61 ^b^	−38.73 ± 0.52 ^b^
Local Market Product	44.48 ± 0.21 ^b^	10.11 ± 0.10 ^a^	11.51 ± 0.17 ^a^	706.97 ± 57.07 ^a^	−27.00 ± 0.08 ^a^

The standard deviation is represented by ±values (*n* = 3). Different letters in each column (according to Tukey test) are statistically significantly different (*p* < 0.05).

**Table 4:** EDS results of cocoa milk without hydrocolloid, optimum sample and local market product.

Products	C (%)	N (%)	O (%)	Na (%)	P (%)	Cl (%)	K (%)	Ca (%)
No hydrocolloid	69.19	7.25	16.50	0.60	0.00	1.30	3.72	1.45
Optimum sample	73.42	5.82	13.08	0.48	0.00	1.49	4.04	1.67
Local Market Product	69.92	6.38	14.54	0.74	0.00	1.99	3.97	2.48

**Table 5:** Nine-month storage results of optimum sample.

Months	WHC (%)	Sedimentation (%)	Creaming (%)	pH	Apparent Viscosity (mPa·s)	Torque (mN·m)
1	92.50 ± 0.71 ^a^	nd	0.012 ± 0.005 ^a^	6.86 ± 0.02 ^a^	59.50 ± 0.55 ^a^	0.269 ± 0.007 ^a^
2	92.40 ± 0.28 ^a^	nd	0.012 ± 0.005 ^a^	6.86 ± 0.01 ^a^	61.72 ± 0.23 ^ab^	0.275 ± 0.010 ^a^
3	92.10 ± 0.14 ^a^	nd	0.016 ± 0.001 ^a^	6.85 ± 0.03 ^a^	63.99 ± 0.16 ^bc^	0.282 ± 0.005 ^a^
4	91.50 ± 0.72 ^a^	nd	0.018 ± 0.002 ^a^	6.83 ± 0.02 ^a^	66.55 ± 0.72 ^cd^	0.294 ± 0.021 ^a^
5	91.05 ± 0.64 ^a^	nd	0.018 ± 0.002 ^a^	6.83 ± 0.01 ^a^	67.48 ± 0.67 ^d^	0.303 ± 0.016 ^a^
6	90.70 ± 0.65 ^a^	nd	0.027 ± 0.005 ^a^	6.80 ± 0.03 ^a^	67.90 ± 0.14 ^d^	0.305 ± 0.023 ^a^
7	90.35 ± 0.21 ^a^	nd	0.033 ± 0.008 ^a^	6.80 ± 0.01 ^a^	68.49 ± 0.31 ^de^	0.308 ± 0.012 ^a^
8	89.50 ± 1.13 ^a^	nd	0.046 ± 0.010 ^a^	6.79 ± 0.02 ^a^	68.65 ± 0.49 ^de^	0.309 ± 0.009 ^a^
9	88.70 ± 0.99 ^a^	nd	0.050 ± 0.010 ^a^	6.79 ± 0.01 ^a^	71.23 ± 0.72 ^e^	0.320 ± 0.018 ^a^

nd: Not detected. The standard deviation is represented by ±values (*n* = 3). Different letters in each column (according to Tukey test) are statistically significantly different (*p* < 0.05).

**Table 6:** Nine-month storage results of local market product.

Months	WHC (%)	Sedimentation (%)	Creaming (%)	pH	Apparent Viscosity (mPa·s)	Torque (mN·m)
1	85.00 ± 1.41 ^a^	4.05 ± 0.14 ^a^	3.20 ± 0.28 ^a^	7.35 ± 0.01	79.75 ± 1.77 ^a^	0.358 ± 0.008 ^a^
2	83.05 ± 1.34 ^a^	7.00 ± 0.28 ^b^	4.80 ± 0.21 ^ab^	7.34 ± 0.03	85.38 ± 2.58 ^ab^	0.382 ± 0.015 ^ab^
3	80.50 ± 0.71 ^ab^	9.33 ± 0.11 ^c^	6.00 ± 0.71 ^bc^	7.31 ± 0.02	92.70 ± 2.26 ^bc^	0.415 ± 0.010 ^abc^
4	79.45 ± 0.92 ^bc^	10.50 ± 0.42 ^cd^	7.00 ± 0.42 ^bcd^	7.30 ± 0.02	98.50 ± 1.56 ^cd^	0.442 ± 0.009 ^bcd^
5	77.55 ± 1.20 ^bc^	11.45 ± 0.64 ^cde^	7.65 ± 0.30 ^cde^	7.28 ± 0.03	103.35 ± 2.76 ^cd^	0.464 ± 0.011 ^cde^
6	74.50 ± 0.57 ^cd^	11.99 ± 0.09 ^de^	8.20 ± 0.64 ^cde^	7.27 ± 0.04	108.90 ± 1.27 ^de^	0.489 ± 0.012 ^de^
7	71.85 ± 0.78 ^de^	12.38 ± 0.18 ^de^	8.48 ± 0.25 ^de^	7.24 ± 0.01	117.49 ± 2.31 ^ef^	0.527 ± 0.007 ^ef^
8	70.55 ± 1.06 ^de^	12.74 ± 0.27 ^e^	8.80 ± 0.16 ^de^	7.23 ± 0.03	127.65 ± 1.77 ^fg^	0.573 ± 0.005 ^fg^
9	68.25 ± 1.20 ^e^	13.00 ± 0.71 ^e^	9.03 ± 0.32 ^e^	7.21 ± 0.02	135.00 ± 2.83 ^g^	0.606 ± 0.024 ^g^

The standard deviation is represented by ±values (*n* = 3). Different letters in each column (according to Tukey test) are statistically significantly different (*p* < 0.05).

## Data Availability

The data presented in this study are available from the corresponding author upon reasonable request.
